# Sagittal imbalance syndrome, a new concept, helps determining a long fusion for patients with degenerative lumbar spinal stenosis and severe global sagittal imbalance

**DOI:** 10.1186/s13018-024-04613-2

**Published:** 2024-02-13

**Authors:** Shibao Lu, Weiguo Zhu, Yu Wang, Chao Kong, Wei Wang, Xiaolong Chen, Xiangyu Li

**Affiliations:** 1 Department of Orthopaedic Surgery, Beijing Xuanwu Hospital, Capital Medical University, Beijing, China; 2 National Geriatric Disease Research Center, Beijing, China

**Keywords:** Sagittal imbalance syndrome, Degenerative lumbar spinal stenosis, Severe spinal sagittal imbalance, Surgical decision making, Spinal deformity

## Abstract

**Objective:**

To retrospectively investigate the postoperative clinical and radiographic outcomes in elderly patients with degenerative lumbar spinal stenosis (DLSS) and severe global sagittal imbalance who underwent different fusion levels.

**Methods:**

A total of 214 patients with DLSS and severe global sagittal imbalance were included. Sagittal imbalance syndrome was defined as the severe decompensated radiographic global sagittal imbalance accompanied with the following symptoms: severe back pain in naturel posture that disappears or significantly relieves in support position, living disability with ODI score > 40% and dynamic sagittal imbalance. Thereinto, 54 patients were found with sagittal imbalance syndrome and were performed the lumbar decompression with a long thoracolumbar fusion (Group A) or a short lumbar fusion (Group B). Thirty patients without sagittal imbalance syndrome who underwent short lumbar decompression and fusion were selected as the control (Group C).

**Results:**

Patients with sagittal imbalance syndrome were detected to have more paraspinal muscle degeneration and less compensatory potentials for sagittal imbalance (smaller thoracic kyphosis and larger pelvic tilt) than those without this diagnosis. Postoperative comparisons revealed significant restoration of global sagittal alignment and balance and improvement of living quality in Groups A and C at the final follow-up. Six patients in Group B and one in Group A were found to have proximal junctional complication during follow-up.

**Conclusion:**

Our results indicated that DLSS patients with sagittal imbalance syndrome had inferior surgical outcomes in terms of living quality and proximal junctional complication after lumbar decompression with a short fusion.

## Introduction

Global spinal sagittal imbalance refers to the spinal malalignment with a significant manifestation of forward postural instability in standing, which is becoming a gradually recognized cause of back pain and disability in adults [1–3]. Previous studies demonstrated that increasing sagittal imbalance was associated with inferior health-related quality-of-life (HRQoL) scores and suboptimal surgical results [4]. Despite secondary to various lesions, sagittal imbalance is common in degenerative lumbar spinal stenosis (DLSS) because of the degenerative changes of disks, vertebrae and paravertebral muscles and the limited compensatory mechanisms. Taking this instability into consideration is necessary when assessing the severity of a DLSS and designing the optimal surgical plan.

However, not all the global spinal sagittal imbalance needs to be corrected. At present, there is a lack of consensus when to simultaneously correct the sagittal imbalance with long fusion in lumbar decompression surgery. Generally, when a severe sagittal imbalance is present, a long fusion from thoracic to lumbar for regulating the global sagittal profile is more likely to be appropriate based on the previous findings that the uncorrected sagittal imbalance following short fusion predisposed inferior surgical outcomes including symptomatic instrumentation failures and revision surgeries [5–7]. Differently, some spinal surgeons would like to perform a short-segment decompression and fusion, regardless of the global imbalance to limit operation time and blood loss [8, 9].

Different from the young, surgical management for the elderly patients with spinal deformity should be mainly focused on relieving symptoms and improving living quality. In the present study, we described a new medical term sagittal imbalance syndrome that summarized the symptomatic conditions of decompensated sagittal imbalance. In our center, DLSS patients with sagittal imbalance syndrome were performed a relative short lumbar decompression and fusion or a long thoracolumbar fusion with lumbar decompression. We conducted this study to investigate the postoperative outcomes after different fusion managements for DLSS patients with severe sagittal imbalance and to explore the fusion level selection strategy.

## Materials and methods

### Subjects

Under the approval from the Ethics Committee of Capital Medical University Xuanwu Hospital (approval number: 2018014), patients with DLSS and severe global sagittal imbalance who received surgical treatment at our center for geriatric diseases from March 2017 to March 2021 were retrospectively reviewed. Patients were exempt from the requirement of informed consent. Sagittal malalignment with PI-LL > 20°, sagittal vertical axis (SVA) > 95 mm or PT > 30° was defined as severe global sagittal imbalance [10]. Inclusion criteria were as follows: (1) aged > 60 years, (2) with a minimum 2-year follow-up, (3) with complete preoperative and postoperative clinical and radiographic data, (4) with a spinal bone mineral density (BMD) T score (Dual-energy X-ray absorptiometry scan) >  − 2.5. Subjects with scoliosis, other sagittal abnormity not associated with degeneration or a surgical history of spine or pelvis were excluded.

## Clinical and radiographic evaluation

### Basic information

**Fig. 1:**
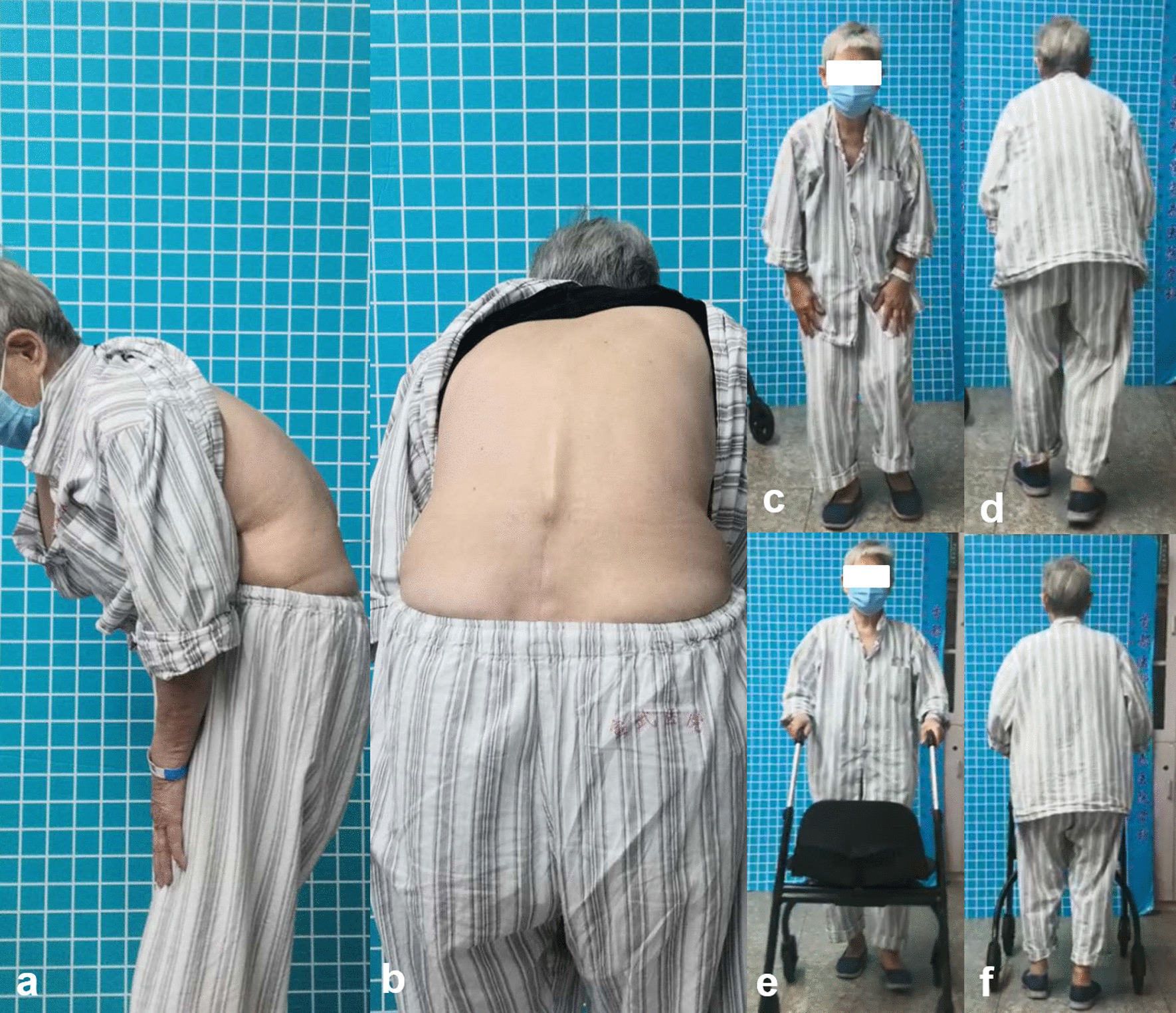
Severe back pain in natural position and comfort with a support. When in natural standing or in natural walking without any support, patient could not maintain an upright position and would complain of a severe back pain (VAS > 5 score) (**a, b, c, d**). Using a walking aid, patient could walk freely and the severe back pain would disappear or significantly relieve (**e, f**)

**Fig. 2:**
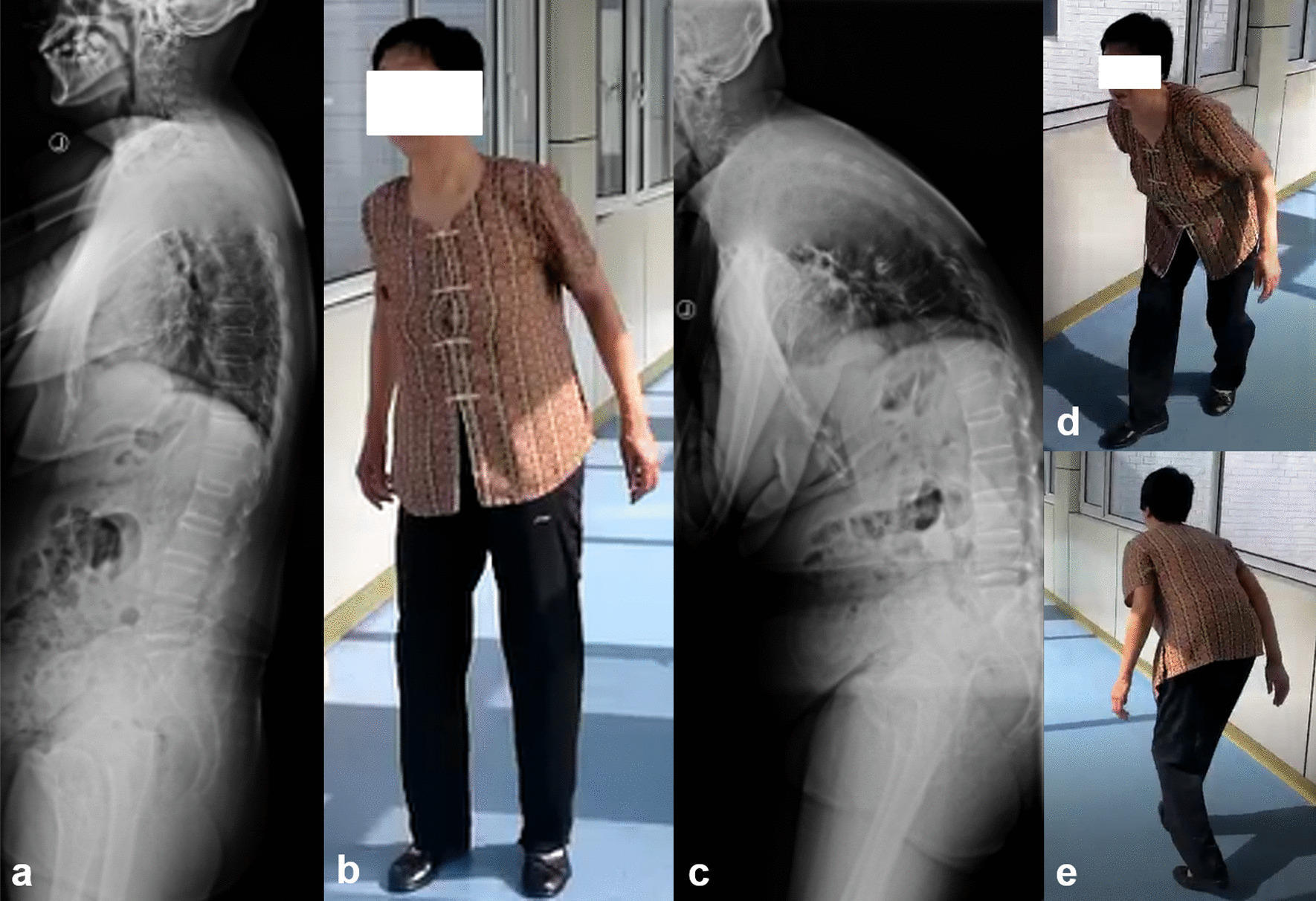
Dynamic sagittal imbalance. Patient could stand upright for a while (**a, b**). After a short walk within 10 min, the compensatory mechanisms exhausted and a significant trunk bent forward appeared (**c, d, e**)

Subjects’ demographic data including age, gender distribution, body mass index (BMI), BMD of lumbar vertebra and surgical information were recorded. At the time of radiographic acquisition, patient-reported outcomes were assessed using visual analogue scale (VAS) and Oswestry disability index (ODI) scores. In the present study, the following symptoms were considered as sagittal imbalance syndrome or symptomatic sagittal imbalance [11]: (1) severe back pain (VAS > 5 score) in natural standing position or in natural walking without any support (Fig. 1a–d). Back pain disappears or significantly relieves in support position (Fig. 1e, f); (2) significant living disability with ODI score > 40% [12, 13] and (3) dynamic sagittal imbalance in walking within 10 min (Fig. 2) [14]. Patients with sagittal imbalance syndrome were randomly performed a long thoracolumbar fusion with lumbar decompression (Group A) or a relative short lumbar decompression and fusion (Group B). In our center, patients with severe global sagittal imbalance without sagittal imbalance syndrome underwent a relative short lumbar decompression and fusion. Thirty of them were randomly selected as control (Group C).

#### Spinopelvic parameters

**Fig. 3:**
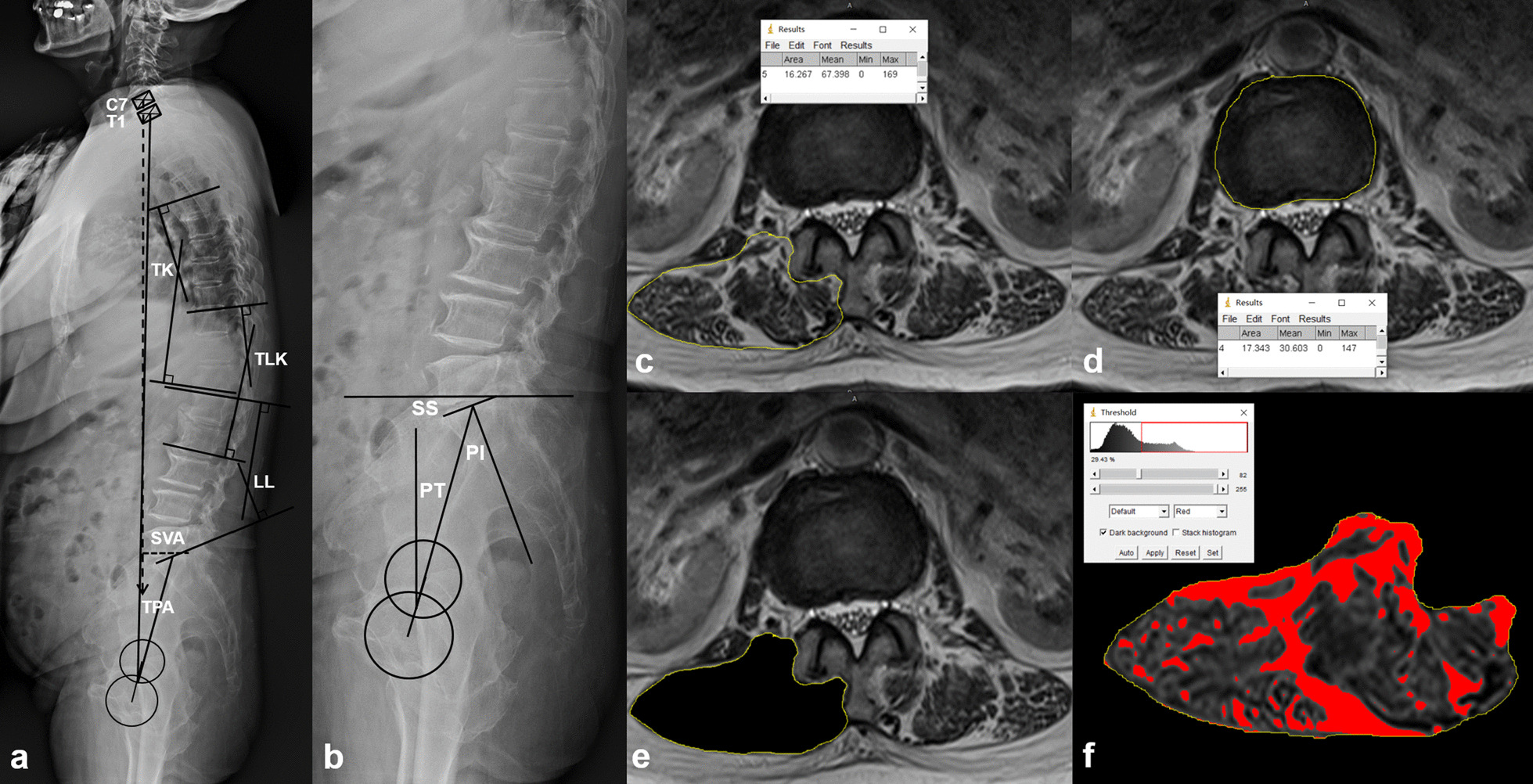
Spinopelvic parameters measurement and back muscle evaluation. **a** Measurement of spinal parameters. **b** Measurement of pelvic parameters. **c** The fascial boundary of lumbar paravertebral muscles (yellow circle): the fascia thoracolumbalis was traced down laterally and anteriorly to the dorsal side of the quadratus lumborum, followed by the posterior surface of the facet and lamina, and lateral margin of spinous process. **d** The boundary of vertebral body (yellow circle). Muscle/disk ratio: 16.267/17.343 = 0.94. **e** Cut the muscle along the fascial boundary. **f** Bright pixels of fat tissue in the MR images were colored in red (darker color in the black and white version) using pseudocoloring technique. The percentage of the red pixel area in the muscle compartment was the percentage of fat infiltration (29.43%)

Radiographic measurements were performed on long-cassette standing upright lateral radiographs of the spine and pelvis. The following radiographic parameters were measured using Surgimap software (Nemaris, Inc., New York, NY, USA) (Fig. 3a, b) [15]: thoracic kyphosis (TK), thoracolumbar kyphosis (TLK), lumbar lordosis (LL), pelvic incidence (PI), sacral slope (SS), pelvic tilt (PT), PI-LL mismatch (PI-LL), sagittal vertical axis (SVA) and T1 pelvic angle (TPA).

#### Disk and facet degeneration evaluations

The degrees of lumbar disk degeneration and right facet arthritis (L1–L2 to L5–S1) were examined on 1.5-T MRI images using Pfirrmann degeneration classification [16]. Different score was given to represent different degeneration grade of disk and facet joint. Higher scores represented better disk and facet conditions. Mean values of the five levels were calculated.

#### Muscle evaluation

Cross-sectional Area of lumbar paravertebral muscle was assessed on 1.5 T MRI images with ImageJ software (National Institutes of Health, Bethesda, Maryland, USA) [17, 18]. T2-weighted axial images at L1–2, L2–3, L3–4 and L4–5 disk levels were analyzed to measure the right muscle area. The regions of interest of back muscle were determined by outlining the fascial boundary of the muscles (Fig. 3c). The signal intensity (in gray scale) within the region of interest was measured using the measurement function of ImageJ. Muscle area was divided by the disk area at the same level (muscle/disk ratio) to decrease the bias caused by individual size (Fig. 3d). The percentage of fat infiltration was measured using a pseudocoloring technique (Fig. 3e, f). Mean values of the four levels were calculated.

At the latest follow-up, patients’ satisfactions of surgical managements were evaluated with centesimal system score from 0 to 100. 0 represents not satisfied, while 100 represents very satisfied. All the clinical and radiographic evaluations were completed by two independent spine surgeons (X.L.C. and X.Y.L.), who were not involved in the treatment of the patients. The mean values were recorded.

### Surgical procedure

The indication for decompression of stenosed lumbar canal was that patients’ neurological symptoms and signs were not obviously resolved after conservative treatment for 6 months. Surgeries were performed by the same team, with pedicle screws and titanium rods. Decompression was completed using transforaminal lumbar interbody fusion (TLIF) technique and posterior instrumentation and fusion through open procedure. In Group A, the proximal end vertebra in the measured kyphosis was selected as the upper instrumented vertebra (UIV). The lowest instrumented vertebra was determined according to the TLIF level. In Groups B and C, instrumented segments were depended on the TLIF levels. During the exposure, the supraspinous ligaments, the interspinous ligaments and the articular processes at UIV level were protected to limit the proximal junctional kyphosis (PJK) complication. In all the groups, the rods would be contoured to restore the lumbar lordosis and restore the sagittal balance. Fusion was finished using autograft and allograft.

### Statistical analysis

Data were analyzed using SPSS version 16.0 statistical software (SPSS Inc.). All data were presented as the mean ± standard deviation. Comparisons between pre-operation and post-operation and between different groups were performed using the Mann–Whitney U test. Chi-square analysis was applied to assess the categorical variables. A *p* value < 0.05 was considered statistically significant.

## Results

### General information

A total of 214 patients (141 females and 73 males) with DLSS and severe global sagittal imbalance were included in this study. Fifty-four patients were found with sagittal imbalance syndrome. Sixteen patients did not reach a minimum 2-year follow-up, including 7 lost to follow-up. Finally, 38 patients were enrolled for the analysis of postoperative outcomes. Thereinto, 18 patients receiving thoracolumbar fusion were assigned to Group A, while 20 patients receiving lumbar fusion were assigned to Group B. During follow-up, 7 cases of PJKs were detected. No patient was observed to have pseudoarthrosis, implant failure or neurological deficits. No revision surgery was required.

### Comparisons of demographic and radiographic characteristics between patients with and without sagittal imbalance syndrome

Patients with sagittal imbalance syndrome had older age than those without this diagnosis (Table 1). Comparisons of spinopelvic parameters revealed statistically smaller TK, LL and SS and statistically greater TLK, PT, PI-LL and TPA in patients with sagittal imbalance syndrome. Except the more muscle fat infiltration in those with sagittal imbalance syndrome, lumbar degenerations were similar between the two groups. As to HRQoL outcomes, patients with sagittal imbalance syndrome were showed to have severer back pain in thoracolumbar region and more significant living disability than those without sagittal imbalance syndrome.

**Table 1:** Comparisons of clinical and preoperative radiographic characteristics between patients with and without sagittal imbalance syndrome

Variables	With sagittal imbalance syndrome (*n* = 54)	Without sagittal imbalance syndrome (*n* = 160)	*P*
Age (year)	66.1 ± 3.9	62.3 ± 4.2	0.007
Gender distribution	Female: 40; Male: 14	Female: 101; Male: 59	0.142^†^
Body mass index (kg/m^2^)	25.5 ± 2.5	26.4 ± 1.6	0.303
Bone mineral density (g/cm2)	1.092 ± 0.084	1.101 ± 0.091	0.321
*Spinopelvic measurements*			
Thoracic kyphosis (°)	12.3 ± 5.0	28.7 ± 4.1	< 0.001
Thoracolumbar kyphosis (°)	16.7 ± 6.2	7.0 ± 2.9	< 0.001
Lumbar lordosis (°)	10.2 ± 7.0	34.6 ± 5.2	< 0.001
Pelvic incidence (°)	49.0 ± 3.9	48.3 ± 4.8	0.596
Pelvic tilt (°)	29.5 ± 4.3	17.2 ± 4.4	< 0.001
Sacral slope (°)	19.7 ± 3.5	32.3 ± 6.3	< 0.001
Pelvic incidence–lumbar lordosis (°)	40.1 ± 4.7	15.0 ± 4.5	< 0.001
Sagittal vertical axis (mm)	115.9 ± 13.5	111.2 ± 18.5	0.088
T1 Pelvic angle (°)	28.8 ± 2.9	22.3 ± 1.8	< 0.001
*Lumbar and muscle degeneration evaluations*			
Disk	2.87 ± 0.15	3.07 ± 0.20	0.132
Facet	2.17 ± 0.13	2.34 ± 0.11	0.082
Muscle/disk ratio	1.34 ± 0.12	1.43 ± 0.12	0.159
Muscle fat infiltration (%)	35.3 ± 3.8	19.5 ± 3.7	< 0.001
Health-related quality of life			
Visual analogue scale (point)	6.6 ± 0.4	4.0 ± 0.4	0.001
Oswestry disability index (%)	55.3 ± 5.4	25.5 ± 4.3	< 0.001

^†^Calculated by Chi-square analysis

### Comparisons of clinical and radiographic assessments between Group A and Group B

As shown in Table 2 and 3, demographic baselines and preoperative radiographic parameters were comparable between the two groups. Patients in Group A were detected to have larger average operation duration, more average estimated blood loss and more average fusion levels than those in Group B (Table 2). After similar follow-up time, TK, TLK, LL, PT, SS, PI-LL, SVA and TPA were all significantly improved in Group A, while only LL, SS and PI-LL were obviously changed in Group B. At the latest follow-up, TK was statistically greater and TLK, PI-LL, SVA and ODI scores were statistically smaller in Group A than Group B (Table 3). Six patients in Group B and one in Group A were found to have PJKs during follow-up. Those underwent the thoracolumbar fusion were more satisfied with their surgical management (84.6 ± 5.9 vs. 63.4 ± 7.2, *P* = 0.003).

**Table 2:** Comparisons of demographic baselines and surgical data between Groups A and B and between Groups B and C

Variables	Group A (*n* = 18)	Group B (*n* = 20)	Group C (*n* = 30)	*P* value	
				*P*_AB_	*P*_BC_
Age (year)	70.4 ± 4.2	68.0 ± 4.8	67.1 ± 5.9	0.302	0.486
Gender	Female: 15; Male: 3	Female: 16; Male: 4	Female: 17; Male: 13	0.791^†^	0.088^†^
Body mass index (kg/m^2^)	24.6 ± 3.2	25.2 ± 3.1	26.7 ± 1.9	0.681	0.442
Bone mineral density (g/cm2)	1.081 ± 0.087	1.103 ± 0.101	1.122 ± 0.099	0.612	0.585
Operation time (min)	342.5 ± 47.2	278.1 ± 63.3	214.6 ± 50.8	0.010	0.141
Estimated blood loss (ml)	746.4 ± 150.6	407.2 ± 84.5	332.9 ± 44.3	0.002	0.372
Fusion levels	9.0 ± 0.6	3.2 ± 0.2	2.7 ± 0.3	< 0.001	0.116
Follow-up (months)	26.4 ± 1.8	27.7 ± 2.3	28.2 ± 1.5	0.149	0.664
Disk degeneration	3.01 ± 0.19	2.90 ± 0.26	3.12 ± 0.23	0.462	0.315
Facet degeneration	2.13 ± 0.15	2.17 ± 0.18	2.38 ± 0.14	0.504	0.261
*Paravertebral muscle degeneration*					
Muscle/disk ratio	1.36 ± 0.15	1.33 ± 0.13	1.37 ± 0.11	0.456	0.363
Fat infiltration (%)	32.2 ± 4.1	36.6 ± 5.2	20.9 ± 4.2	0.340	< 0.001

Group A includes the patients with DLSS and sagittal imbalance syndrome who underwent a thoracolumbar fusion including lumbar decompression and global sagittal restoration, Group B includes the patients with DLSS and sagittal imbalance syndrome who underwent lumbar decompression and fusion, and Group C includes patients with DLSS and severe sagittal deformity without sagittal imbalance syndrome who underwent lumbar decompression and fusion

^†^Calculated by Chi-square analysis

**Table 3:** Comparisons of preoperative and postoperative radiographic measurements and HRQOL outcomes between Groups A and B and between Groups B and C

Variables	Group A (*n* = 18)	Group B (*n* = 20)	Group C (*n* = 30)	*P* value	
				*P*_AB_	*P*_BC_
*Thoracic kyphosis (°)*					
Preoperatively	10.7 ± 5.4	12.4 ± 6.3	30.8 ± 5.4	0.515	< 0.001
At the latest follow-up	22.0 ± 7.6	15.8 ± 5.0	32.5 ± 3.7	0.022	0.003
*P* value	0.033	0.319	0.446	–	–
*Thoracolumbar kyphosis (°)*					
Preoperatively	19.3 ± 6.5	17.7 ± 9.0	11.8 ± 4.1	0.391	0.060
At the latest follow-up	4.7 ± 3.6	12.9 ± 7.8	5.0 ± 4.4	< 0.001	0.014
*P* value	0.010	0.209	0.366	–	–
*Lumbar lordosis (°)*					
Preoperatively	5.8 ± 9.2	5.2 ± 7.4	20.5 ± 6.9	0.524	0.001
At the latest follow-up	41.5 ± 8.3	39.0 ± 6.8	40 ± 5.2	0.512	0.660
*P* value	< 0.001	< 0.001	0.001	–	–
*Pelvic incidence (°)*					
Preoperatively	47.7 ± 4.1	49.2 ± 5.7	49.4 ± 6.2	0.483	0.499
At the latest follow-up	51.2 ± 6.3	50.4 ± 5.2	47.7 ± 4.5	0.491	0.605
*P* value	0.102	0.283	0.329	–	–
*Pelvic tilt (°)*					
Preoperatively	29.5 ± 5.7	29.2 ± 6.5	15.3 ± 5.3	0.601	0.001
At the latest follow-up	20.6 ± 6.9	23.1 ± 7.3	9.8 ± 7.2	0.272	< 0.001
*P* value	0.044	0.103	0.112	–	
*Sacral slope (°)*					
Preoperatively	17.4 ± 7.1	19.3 ± 3.7	34.9 ± 7.5	0.366	0.021
At the latest follow-up	31.3 ± 8.9	28.6 ± 5.5	40.8 ± 6.2	0.280	0.098
*P* value	0.009	0.030	0.443	–	
*Pelvic incidence–lumbar lordosis (°)*					
Preoperatively	41.5 ± 6.3	42.4 ± 6.0	27.2 ± 6.4	0.407	< 0.001
At the latest follow-up	8.5 ± 5.2	19.8 ± 5.5	7.3 ± 4.8	0.001	0.002
*P* value	< 0.001	0.001	0.014	–	–
*Sagittal vertical axis (mm)*					
Preoperatively	125.3 ± 16.1	120.7 ± 24.4	108.2 ± 18.5	0.529	0.106
At the latest follow-up	32.7 ± 7.2	93.5 ± 24.0	31.5 ± 9.3	< 0.001	< 0.001
*P* value	< 0.001	0.077	< 0.001	–	–
*T1 Pelvic angle (°)*					
Preoperatively	30.2 ± 3.1	29.0 ± 4.7	27.6 ± 2.1	0.614	0.422
At the latest follow-up	16.7 ± 4.4	22.3 ± 5.3	16.0 ± 3.3	0.191	0.184
*P* value	0.036	0.113	0.074	–	–
*Visual analogue scale (point)*					
Preoperatively	6.9 ± 0.5	6.5 ± 0.7	4.2 ± 0.4	0.701	0.026
At the latest follow-up	2.4 ± 0.8	3.8 ± 0.5	1.3 ± 0.5	0.101	0.003
*P* value	0.009	0.084	< 0.001	–	–
*Oswestry disability index (%)*					
Preoperatively	59.5 ± 7.0	56.3 ± 5.6	28.1 ± 4.6	0.214	< 0.001
At the latest follow-up	28.7 ± 6.4	40.3 ± 10.8	22.4 ± 6.5	0.011	< 0.001
*P* value	< 0.001	0.057	0.168	–	–

Group A includes the patients with DLSS and sagittal imbalance syndrome who underwent a thoracolumbar fusion including lumbar decompression and global sagittal restoration, Group B includes the patients with DLSS and sagittal imbalance syndrome who underwent lumbar decompression and fusion, Group C includes patients with DLSS and severe sagittal deformity without sagittal imbalance syndrome who underwent lumbar decompression and fusion

### Comparisons of clinical and radiographic assessments between Group B and Group C

The two groups were matched in terms of demographic baselines, surgical data and lumbar degenerations (Table 2). Patients with sagittal imbalance syndrome in Group B were found to have more significant degeneration of back muscle and inferior patient-reported outcomes than those without sagittal imbalance syndrome in Group C. TK, LL and SS were significantly smaller, whereas TLK, PT and PI-LL were statistically greater in Group B (Table 3). Despite with the same surgical procedure, the postoperative outcomes regarding the restoration of global sagittal balance were distinct: SVA was decreased from 108.2 ± 18.5 mm to 31.5 ± 9.3 mm (*P* < 0.001) in Group C, whereas was not significantly changed in Group B (Table 3). At the latest follow-up, no complication at the proximal segment was found in Group C. Patients’ postoperative self-reported scores and satisfactions of their surgical treatment were superior in Group C than Group B.

## Discussion

Ideal global spinal alignment allows an individual to assume an upright posture with minimal muscular energy expenditure. Increasing positive global sagittal imbalance will add the trunk muscular effort and energy expenditure, which can result in muscular back pain, fatigue and even living disability [19]. The operative treatment of sagittal imbalance is complex and potentially associated with significant complications, especially in the elderly population. We previously discovered severe back pain (VAS > 5 score), significant living disability (ODI > 40%) and dynamic sagittal imbalance were the risk factors of suboptimal postoperative outcomes in patients with DLSS and severe global sagittal imbalance after short lumbar fusion [11]. In this study, severe back pain without support (Fig. 1), significant living disability and dynamic sagittal imbalance (Fig. 2) were considered as sagittal imbalance syndrome or symptomatic sagittal imbalance. Comparisons between patients with and without sagittal imbalance syndrome revealed that those with sagittal imbalance syndrome had more fat infiltration in lumbar muscle (35.3 ± 3.8% vs. 19.5 ± 3.7%, *P* < 0.001, Table 1). The clinical importance of trunk muscle on quality of life and upright posture have been well documented [20, 21]. Paraspinal muscle plays an essential role in spine compensating for sagittal imbalance. High-quality muscle had the power to maintain an upright position with no or minor muscular back pain. It would be hard for dysfunctional spinal muscle to compensate for the severe sagittal imbalance. Besides, patients with sagittal imbalance syndrome were revealed to have smaller TK and SS, which indicated the insufficient potentials for sagittal compensation (Table 1). When the compensatory mechanisms exhausted, patients would present dynamic sagittal instability in walking and develop the related symptoms (Fig. 2).

**Fig. 4:**
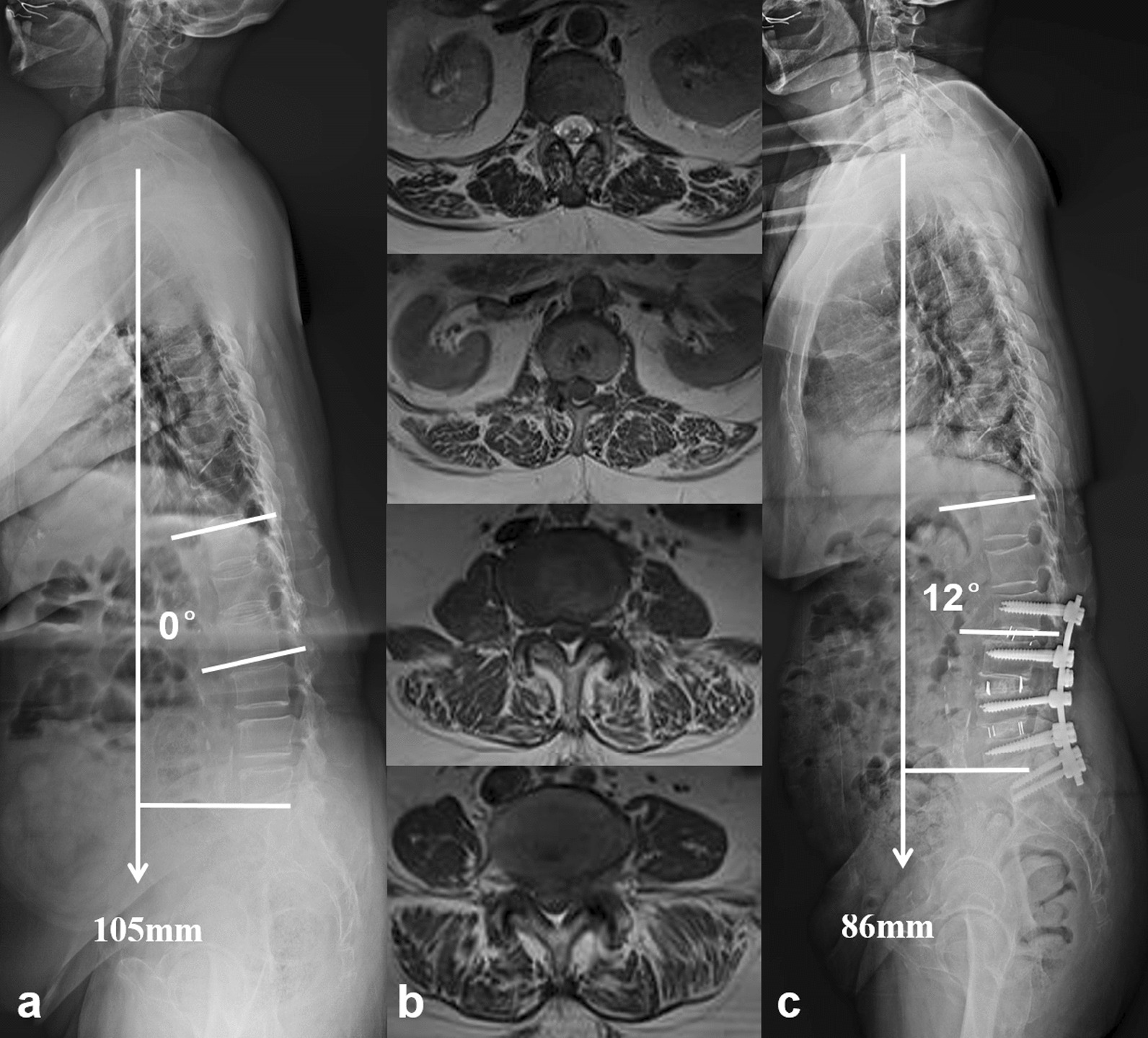
**a** A 68-year-old female patient diagnosed with DLSS and sagittal imbalance syndrome. **b** Paravertebral muscle from L1–2 to L4–5 were infiltrated with fat, with a mean percentage of 36.0% and a mean muscle/disk ratio of 1.41. **c** Twenty-five months after a short-segment lumbar decompression and fusion from L2 to S1, sagittal imbalance was not substantially modified. A complication of PJK was observed

Gilad et al. [7] retrospectively reviewed the surgical outcomes in 47 patients with sagittal plane deformity and found those with uncorrected sagittal imbalance were more likely to develop symptomatic instrumentation failure over a 2-year period. Hori et al. [22] reported that postoperative sagittal decompensation significantly impacts the surgical outcomes of short fusion for DLSS after at least 2-year follow-up. Our preliminary investigation also revealed that patients with postoperative sagittal decompensation were susceptible to PJKs after short lumbar fusion [11]. Uncorrected sagittal imbalance would increase the stress concentration at the proximal adjacent segment, which then induced the development of mechanical complications. Paraspinal musculature deterioration was also demonstrated to be an important and existing risk factor of PJK after spinal fusion for adult spinal deformity [23, 24]. This study presented the consistent result that SVA and TPA in patients with sagittal imbalance syndrome who underwent relatively short lumbar fusion were not significantly modified after more than 2-year follow-up. As a result, 30% of them (6/20) were detected to have PJK complication (Fig. 4), which contributed to their unconspicuous improvement of living quality and low satisfaction of surgical management. To sum up, a relative short lumbar fusion was not adequate for DLSS with sagittal imbalance syndrome.

**Fig. 5:**
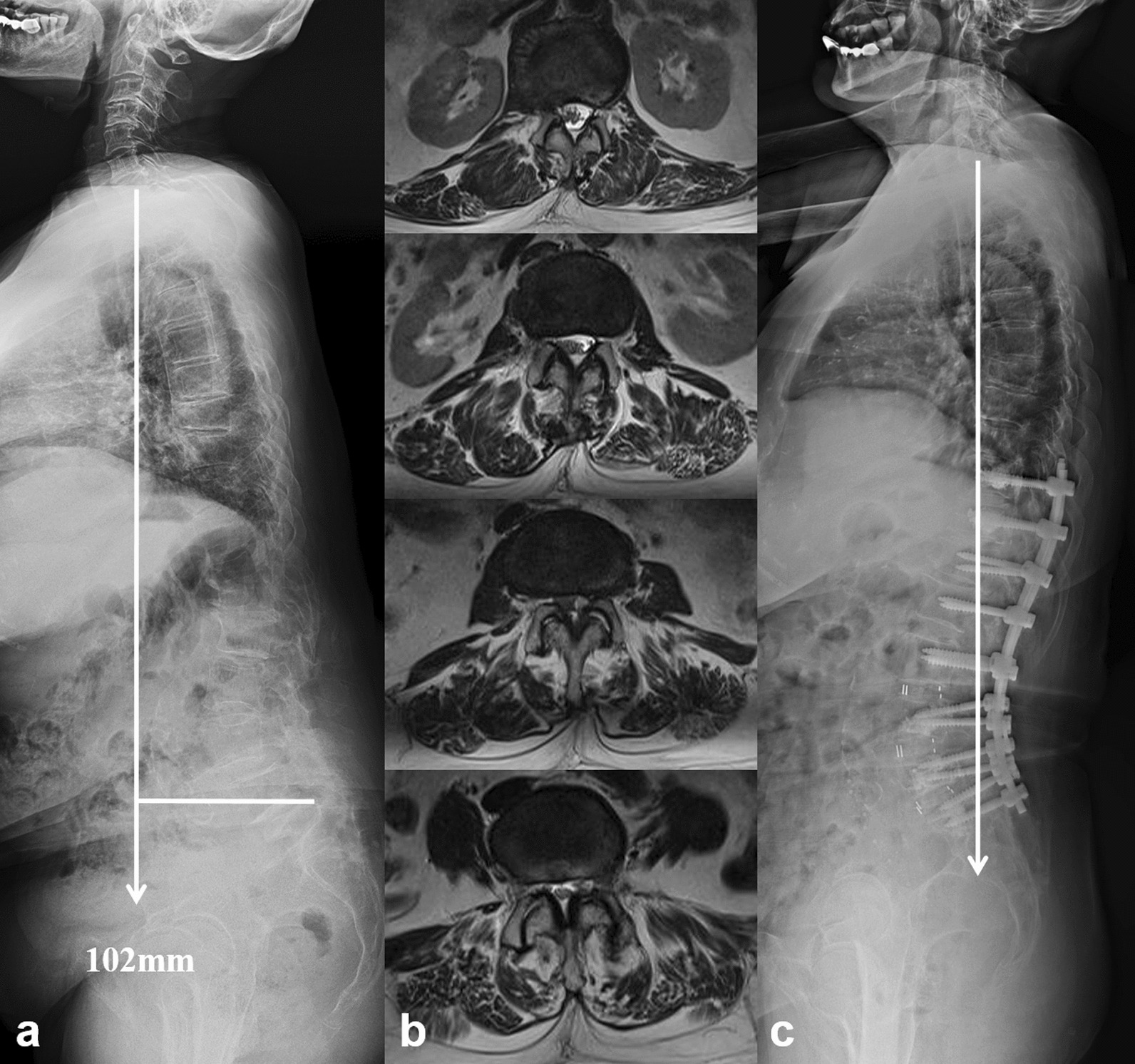
**a** A 68-year-old female patient with DLSS and sagittal imbalance syndrome. **b** Paravertebral muscles from L1–2 to L4–5 were featured with fatty infiltration, with a mean infiltrated percentage of 32.86% and a mean muscle/disk ratio of 1.46. **c** A long fusion from T11 to S1 with lumbar decompression and sagittal realignment was performed. At 24-month follow-up, the global sagittal malalignment and imbalance were completely regulated, without any mechanical complication

In this study, 18 patients with DLSS and sagittal imbalance syndrome underwent a thoracolumbar fusion to simultaneously decompress the stenosed canal and realign the sagittal profile. After 2-year follow-up, the significant symptoms were resolved and their living quality was obviously improved (Table 2, Fig. 5). Previous studies also reported the importance of sagittal imbalance correction on the improvement of patients’ quality of life. Savage and Patel [25] reviewed the evaluation and management of fixed sagittal plane imbalance and concluded that fixed sagittal malalignment often required surgical reconstructive procedures. Reestablishing harmonious spinopelvic alignment was associated with significant improvement in HRQoL outcomes and patients’ satisfaction. Lee et al. [26] conducted a meta-analysis including 327 adult patients from 10 studies on the efficacy of surgical correction of PDSI. They drew a conclusion that the restoration of global sagittal alignment was essential for relieving back pain and improving patients’ living quality.

**Fig. 6:**
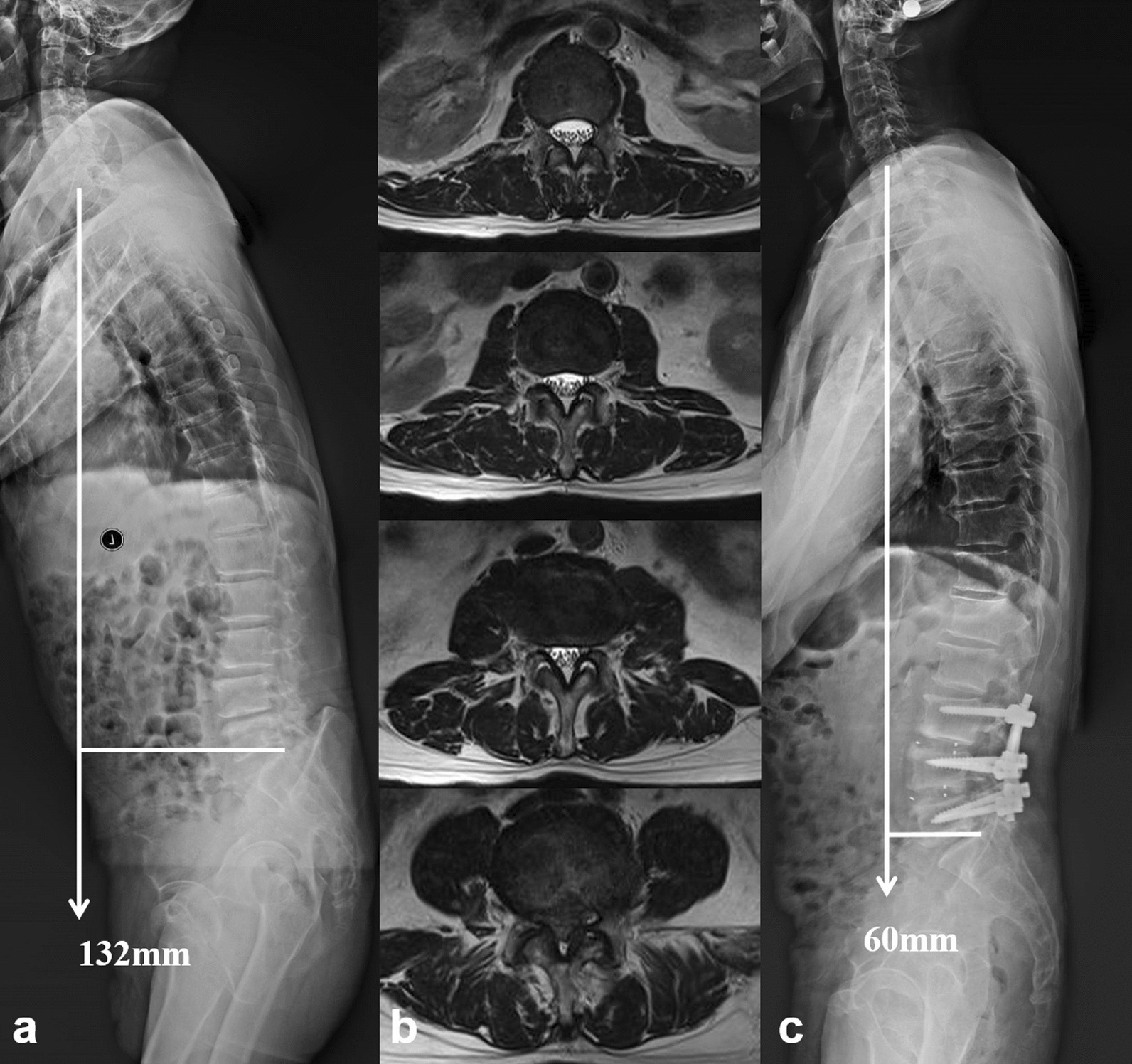
**a** A 66-year-old male patient with DLSS and PDSI who was not diagnosed with sagittal imbalance syndrome. **b** He had a good lumbar muscle status. The mean muscle/disk ratio was 1.35 and the mean percentage of fatty infiltrated was 19.4%. **c** Two years after lumbar decompression and short fusion, his global sagittal imbalance was spontaneously compensated. No internal-related untoward event was revealed

However, not all the global spinal sagittal deformity is needed to be corrected. If patients only have radiographic sagittal deformity without the clinical symptoms, their global imbalance might be a temporary lenitive or relieving posture for low back pain that is associated with spinal stenosis. Hence, correcting the global malalignment with thoracolumbar fusion might be an overtreatment. The present study discovered that patients in Group C who underwent lumbar decompression and fusion obtained a satisfied spontaneous restoration of global sagittal balance at the final follow-up, with SVA decreased from 108.2 ± 18.5 to 31.5 ± 9.3 mm (Table 3, Fig. 6). We deemed their substantial quality of paraspinal muscle contributed to the optimistic results (Table 2). As the posterior tension band of spine, trunk muscle played an important role in compensating for sagittal imbalance [20]. The functional back muscle had the ability to self-regulate the sagittal alignment and self-restore the sagittal balance after short lumbar fusion.

Severe sagittal imbalance could cause significant pain and functional limitations. The surgical procedures are potentially associated with a relatively high rate of untoward events and suboptimal outcomes. A reasonable indication for correcting sagittal imbalance could benefit patients from operation more than loss. Surgical intervention for degenerative spinal deformity in elderly should focus on relieving related symptoms. Sagittal imbalance syndrome, summarizing the related symptoms attributable to decompensated sagittal deformity, was demonstrated to have poor paraspinal muscle quality and limited compensatory ability, which were not a reasonable indication for short lumbar fusion. The present findings could help designing a superior surgical plan for elderly patients who suffer from both DLSS and severe sagittal deformity.

Despite, this study still has some limitations. First, this study was a retrospective design with a possible selection bias. The final surgical option for DLSS and sagittal imbalance syndrome was determined by patients and their relatives. Although the clinical and radiographic data were comparable between Group A and Group B, we could not deny the possibility that their pathogenesis of sagittal imbalance was unhomogeneous. Second, the sample size of patients with DLSS and sagittal imbalance syndrome was relatively small, because we excluded all the subjects with any other sagittal spinal anomaly that was not associated with degeneration. Third, thoracic paravertebral muscle was not evaluated. Most of the patients with DLSS were not performed thoracic MRI in our center; therefore, we only focused on the lumbar paravertebral muscle. Fourth, follow-up time was relative short. Despite these, the new term sagittal imbalance syndrome could play an important role in the fusion level decision making for PDSI in elderly DLSS patients.

## Conclusion

Our results indicated that DLSS patients with sagittal imbalance syndrome had inferior surgical outcomes in terms of living quality and proximal junctional complication after lumbar decompression with a short fusion. For patients without sagittal imbalance syndrome, short lumbar decompression and fusion might be an adequate option.
